# Smoking is associated with pessimistic and avoidant beliefs about cancer: results from the International Cancer Benchmarking Partnership

**DOI:** 10.1038/bjc.2015.148

**Published:** 2015-05-07

**Authors:** S L Quaife, A McEwen, S M Janes, J Wardle

**Affiliations:** 1 Health Behaviour Research Centre, Department of Epidemiology and Public Health, University College London, Gower Street, London WC1E 6BT, UK; 2 Lungs for Living Research Centre, UCL Respiratory, Division of Medicine, Rayne Building, University College London, 5 University Street, London WC1E 6JF, UK

**Keywords:** smoking, cancer, attitudes, early detection, avoidance, pessimism

## Abstract

**Background:**

Smoking cessation is the key cancer prevention behaviour for smokers; nonetheless, smokers can still benefit from earlier diagnosis of cancer. However, fewer smokers participate in screening despite their increased risk, which may reflect different beliefs about cancer.

**Methods:**

A UK population-representative sample of ⩾50 year-olds (*n*=6965) was surveyed using the Awareness and Beliefs about Cancer measure. These analyses examine six items on cancer beliefs (e.g., ‘cancer can often be cured'), and four on help-seeking barriers (e.g., ‘I would be too embarrassed').

**Results:**

Smokers were more likely to hold pessimistic cancer beliefs than never-smokers or former-smokers on four of six items. For example, 34% agreed ‘a cancer diagnosis is a death sentence', compared with 24% of non/former-smokers (*P*<0.001). More smokers (18%) than non/former-smokers (11%) would not want to know if they had cancer (*P*<0.01). The only barrier to symptomatic help-seeking differing by smoking status was ‘worry about what the doctor might find' (36% *vs* 28%, *P*<0.01). Associations were independent of demographics, self-rated health and cancer experience.

**Conclusions:**

Smokers held more pessimistic and avoidant beliefs about cancer, which could deter early-detection behaviour. A better understanding of these beliefs is needed to increase engagement in early diagnosis by this high-risk group.

Almost 20% of all new cancer diagnoses each year in the UK, rising to 86% of lung cancer diagnoses, are caused by smoking (Parkin, 2011). Smoking is not only the key risk factor for lung cancer but has also been linked to cancer risk at multiple sites, including the colon, rectum, and uterine cervix; and evidence is emerging for a role in breast cancer (Secretan *et al*, 2009). Smoking cessation is therefore the most important cancer prevention behaviour for smokers. However, smokers can also benefit from screening for colorectal, cervical and breast cancer, and from prompt help-seeking for any potential cancer symptoms.

In comparison with their non-smoking counterparts, smokers are less likely to have been screened for breast, colorectal or cervical cancer, and among those who have been screened, smokers are less likely to meet current recommendations, independent of socioeconomic status (Fredman *et al*, 1999; Sutton *et al*, 2000; Byrne *et al*, 2010, 2014; Vander Weg *et al*, 2012). Smokers show less interest in the prospect of lung cancer screening (Silvestri *et al*, 2007), and delay longer before presenting to their GP with warning signs for lung cancer (Corner *et al*, 2006), particularly those who have been lifelong smokers, have chronic illnesses, or live alone (Smith *et al*, 2009). There is also some indication in the literature that smokers avoid contact with primary care more generally (Kannan and Veazie, 2014), and two studies suggest that smoking is associated with an increased time to help-seeking for cancer types other than lung (Hansen *et al*, 2008; Innos *et al*, 2013). Additionally, studies have found smokers were less likely to believe that mammograms provide peace of mind or were necessary in the absence of symptoms (Messina *et al*, 2002), and they perceived cervical cancer screening to be less important than non-smokers (Marteau *et al*, 2002). Ultimately, poorer engagement in early detection is likely to exacerbate smokers' increased risk of death from cancer, and will mean they miss out on potential teachable moments in smoking cessation.

The fact that fewer smokers engage in early-detection behaviours despite their increased risk is an important paradox to understand. The effect seems to be independent of sociodemographic characteristics (Byrne *et al*, 2010; Vander Weg *et al*, 2012), but attitudinal factors may be important (von Wagner *et al*, 2011). Research to date has focused predominantly on attitudes to lung cancer. A number of clinical studies investigating reasons for delayed help-seeking in smokers who have been diagnosed with lung cancer implicate misattribution of symptoms (Corner *et al*, 2006), and perceived stigma and blame (Tod *et al*, 2008; Chatwin and Sanders, 2013). Fatalistic attitudes (the belief that cancer is neither preventable nor survivable) also appear to be more common in smokers (Niederdeppe and Levy, 2007), and are reported more frequently by cancer patients who continue smoking after their diagnosis than in those who quit (Schnoll *et al*, 2002; McBride and Ostroff, 2003), as well as by individuals who decline the offer of lung cancer screening (Patel *et al*, 2012). However, because fatalism is associated with both deprivation and smoking, it is unclear whether fatalistic perceptions are better explained by socioeconomic circumstances or smoking status.

There have been no large-scale quantitative studies that examine smokers' attitudes towards cancer more generally. We therefore used data from a large population-based survey to compare beliefs about cancer, early diagnosis, and help-seeking for symptoms in current-smokers compared with former-smokers and never-smokers.

## Materials and methods

Data were collected in 2011 as part of Module 2 of the International Cancer Benchmarking Partnership (ICBP). Adults aged ⩾50 years from six countries (United Kingdom, Australia, Canada, Denmark, Sweden, and Norway) were surveyed to provide an international comparison of Awareness and Beliefs about Cancer (ABC) (Forbes *et al*, 2013). The present analyses focus on the UK data concerning beliefs about cancer outcomes, early detection, and barriers to help-seeking.

Random probability sampling methods were used to select households from electronic listings of ‘landline' telephone numbers. Before dialling, the last two digits of each telephone number were replaced with two random digits, to include non-listed numbers. If two or more adults from the same household were eligible for telephone interview (age ⩾50 years), the ‘Rizzo' method was used to randomly select one person to take part (Rizzo *et al*, 2004).

### Awareness and Beliefs about Cancer Measure

The ABC was completed during a computer-assisted telephone interview. ABC items were adapted from previous measures (Paul *et al*, 2006; Stubbings *et al*, 2009; Park and Clery, 2010) for the purpose of telephone interview, and have acceptable psychometric properties (Simon *et al*, 2012).

Beliefs about cancer outcomes and early detection were assessed with six items: (‘A cancer diagnosis is a death sentence' ‘I would not want to know if I have cancer' ‘These days, many people with cancer can expect to continue with normal activities and responsibilities' ‘Cancer can often be cured' ‘Going to the doctor as quickly as possible after noticing a symptom of cancer could increase the chances of surviving' ‘Most cancer treatment is worse than the cancer itself'). Response options were strongly agree, tend to agree, tend to disagree, and strongly disagree; with ‘don't know' responses also recorded. For most analyses, we dichotomised responses as strongly agree/agree *vs* strongly disagree/disagree/don't know.

Four items assessed perceived barriers to help-seeking for a symptom the person thought could be serious: (i) ‘I would be worried about what the doctor might find', (ii) ‘I would be worried about wasting the doctor's time', (iii) ‘I would be too embarrassed', and (iv) ‘I am too busy to make time to go to the doctor'. Interviewers explained, ‘Sometimes people put off going to the doctor even when they have a symptom they think might be serious. These are some of the reasons people give for delaying. Could you say if any of these might put you off going to the doctor?' Response options were yes often, yes sometimes, no, and don't know. The first and last two responses were combined for analysis.

Smoking status was categorised as current, former, or never, on the basis of respondents' answers to two questions: ‘Do you smoke at all these days, either cigarettes, including hand-rolled ones, pipes or cigars?' was used to classify current-smokers, and ‘Have you ever smoked either cigarettes, including hand-rolled ones, pipes or cigars?' was used to identify former-smokers. Those answering no to both questions were considered never-smokers.

Demographic characteristics included age, gender, ethnicity (white *vs* other ethnicity), marital status (married/cohabiting *vs* single/divorced/separated/widowed), highest level of education (left school age ⩽15; CSEs, O-levels or equivalent; A-levels, further education or equivalent; university degree), and three home countries within the United Kingdom (England, Wales, Northern Ireland). As a measure of health status, participants were asked, ‘In general, would you say your health is…?' following which they could choose very good, good, fair, poor, or very poor. For analyses, responses were coded as very good/good *vs* fair *vs* very poor/poor. Experience of cancer, either personally or in close others, was assessed and coded as yes (self or close other) *vs* no for the present analyses.

### Analyses

*χ*^2^ analyses were used to investigate associations between smoking status and the endorsement of each of the belief and help-seeking barrier items. Multivariable logistic regression modelling was used to explore the independence of these associations. Models were run predicting the odds of a positive response to each belief or barrier item by smoking status, and adjusting for age, sex, marital status, ethnicity, region, highest level of education, self-rated health, and cancer experience. The reference group was those who did not agree (i.e., answered ‘disagree', ‘no' or ‘don't know'). For the majority of the belief items (5/6), ‘don't know' responses were relatively uncommon (ranging from 0.5 to 4.3%). The exception was the belief that, ‘most cancer treatment is worse than the cancer itself' to which 15.6% of participants responded ‘don't know'. Therefore, sensitivity analyses were repeated for the belief items excluding cases answering ‘don't know' on any of the belief items (*n*=5138). These were not carried out for the barrier items, because ‘don't know' responses were infrequent (⩽0.3%). Refused responses were minimal across all of the belief and barrier items (⩽0.3%) and were excluded from the analyses.

## Results

Interviewers contacted 24 231 households in England, Wales and Northern Ireland, identifying 10  977 adults eligible for interview (age ⩾50 years). A total of 6965 adults completed the full interview. The overall response rate was calculated as 19.5% using estimates of non-respondent eligibility for those households, which could not be contacted or assessed for eligibility. These estimates were based on the proportion of eligible households for which eligibility was assessed (AAPOR Response Rate 3 conventions; The American Association of Public Opinion Research, 2011). Further details on response rates are available in the main ICBP report (Forbes *et al*, 2013).

Table 1 presents the demographic characteristics of the sample by smoking status. Current-smokers comprised 15% of the overall sample, former-smokers 39%, and never-smokers 46%. In comparison with never- or former-smokers, current-smokers were younger, less likely to be married, less educated, and rated their health as poorer, which mirrors the characteristics of smokers in the general population (ONS, 2011).

### Beliefs about cancer outcomes and early detection

For four of the six cancer beliefs, current-smokers were more pessimistic about cancer outcomes and early detection than former- or never-smokers (see Table 2). The absolute difference in agreement by smoking status was largest for the belief that a ‘cancer diagnosis is a death sentence', with 34% of current-smokers agreeing, compared with 24% of never- or former-smokers. More current-smokers also agreed that ‘I would not want to know if I have cancer' (18% *vs* 11% of both non-smoking groups). Fewer current-smokers agreed that ‘many people with cancer can expect to continue with normal activities and responsibilities' than never- or former-smokers (82% *vs* 90% and 88%, respectively), or that ‘cancer can often be cured' (87% *vs* 91% and 90%). These associations remained significant in multivariable analyses adjusting for demographics, self-rated health and cancer experience, with significantly different odds of agreement in current-smokers compared with never-smokers for the belief that cancer is a death sentence (OR=1.55, 1.32–1.82), that cancer can often be cured (OR=0.73, 0.58–0.92), that they would not want to know if they have cancer (OR=1.44; CI: 1.17–1.78) and the ability to continue with normal activities following a cancer diagnosis (OR=0.54, 0.44–0.67). Strikingly, the responses of former-smokers largely resembled those of never-smokers across all the domains. The absolute difference between their responses was no more than 2.3%.

Smoking status did not affect the odds of believing that ‘going to the doctor quickly increases chances of surviving', which was endorsed almost unanimously (⩾97%). It was also not related to agreeing that ‘cancer treatment is worse than the cancer'.

### Barriers to help-seeking

Current-smokers were more likely to say that being ‘worried about what the doctor might find' might put them off going to the doctor: 36% *vs* 28% of former/never-smokers. In multivariate analyses, this association was independent of demographics, self-rated health and cancer experience, with current-smokers' odds of agreement significantly increased compared with never-smokers (OR=1.25, 1.07–1.47; see Table 3). Current- and former-smokers were less likely than never-smokers to think that being ‘too embarrassed' would deter them from help-seeking (OR=0.79, 0.65–0.97 and OR=0.81, 0.69–0.93, respectively), although the absolute differences in agreement were small (⩽3.7%). Otherwise, there were marginal differences between groups in the likelihood of endorsing the other two barrier items concerning being too busy and worry about wasting the doctor's time.

## Discussion

This is the first population-based study to explore smokers' beliefs about outcomes for cancer in general, with the aim of shedding light on why fewer smokers participate in early detection across a range of cancer types (Silvestri *et al*, 2007; Smith *et al*, 2009; Byrne *et al*, 2010, 2014; Vander Weg *et al*, 2012; Innos *et al*, 2013). We found that smokers held more negative perceptions of cancer outcomes, independent of demographic characteristics, self-rated health and cancer experience. In comparison with non-smokers, they were more likely to believe cancer is a death sentence, saw less chance of cure, and were less likely to believe that normal activities can be continued. They were also less likely to want to know if they have cancer, although they were no less likely to agree with the early-detection principle that prompt presentation increases the chance of survival.

Barriers to help-seeking that concerned embarrassment or time issues (doctor's or own time) differed little by smoking status. But worry about ‘what the doctor might find' was a stronger barrier to help-seeking for current-smokers than never- or former-smokers. Paradoxically, this suggests that smokers' awareness and concern about their poor health is instrumental in deterring early detection.

The present findings suggest that the fatalism about lung cancer that has been observed in smokers (Schnoll *et al*, 2002; McBride and Ostroff, 2003; Kerr *et al*, 2006; Tod *et al*, 2008) may be part of a more generalised negative view of cancer outcomes in the ‘well' smoking population. This might help explain why more smokers in this sample would prefer to remain oblivious if they had cancer, and would avoid going to the doctor if they had symptoms; beliefs consistent with their lower participation in screening (Corner *et al*, 2006; Smith *et al*, 2009; Vander Weg *et al*, 2012). Knowing one's diagnosis might appear to be of little value if the outcome is assumed to be invariably negative. Furthermore, the majority of smokers are aware that smoking adversely impacts health (Siahpush *et al*, 2006) and could be deterred by concern that a diagnosis would mean they would have to quit.

Smokers' pessimism about cancer outcomes may derive partly from their experience of the disease in their friends and family; although smokers in the present sample did not report greater cancer experience than former- or never-smokers. Smokers have poorer health, are overrepresented in socioeconomically deprived groups, and know more smokers, all of which increase the likelihood of experiencing poor cancer outcomes in their social network. Although our analyses controlled for education, self-rated health, and experience of cancer, more sensitive measures of the type of cancer experience (i.e., positive or negative), as well as details of current health problems, would help to further explore the viability of this explanation. However, the fact that current-smokers were more pessimistic than both former- and never-smokers militates against this explanation, as former-smokers are likely to have similar experiential influences given that they were, of course, once smokers themselves.

Alternatively, pessimism might come from being dependent upon tobacco while knowing that it is the most widely recognised cancer risk. This could foster a sense of helplessness over the extent to which health can be controlled; consistent with evidence that female smokers are less likely to believe mammograms provide a sense of control over health (Messina *et al*, 2002). In this context, fatalistic beliefs could help smokers cope with the uncertain health consequences of their habit (Keeley *et al*, 2009); particularly older smokers who are likely to have accrued a significant smoking history and fear that the damage is already done. In other words, both fatalistic and avoidant beliefs could help to relieve the cognitive dissonance experienced from knowing that smoking could cause cancer, but feeling powerless to quit (Festinger, 1957). Having been unable to change their behaviour in the past, they change their beliefs (Gibbons *et al*, 1997; Fotuhi *et al*, 2013).

Our finding that former-smokers' beliefs were more like never-smokers than current-smokers is consistent with US data on attitudes towards lung cancer screening specifically (Silvestri *et al*, 2007). Together, they suggest that negativity about cancer is not a stable characteristic of those who take up smoking, but is instead conditional on current behaviour. In support, longitudinal data suggest that smokers' tendency to agree with risk-minimising health beliefs varies with quit attempts (Fotuhi *et al*, 2013). It is possible that successfully quitting smoking fosters a greater sense of optimism and control over health, perhaps by relieving the helplessness and cognitive dissonance former-smokers may have felt when smoking. Another possible explanation is that those who continue to smoke into older adulthood are a more ‘hardcore' group who regard smoking as a positive part of their identity, and as such hold different attitudes to those who have quit (Jarvis *et al*, 2003; Tombor *et al*, 2013). These results indicate that helping smokers to quit may ultimately not only reduce health risks directly but also indirectly increase utilisation of preventive or early-detection opportunities, and this should remain the priority for finite health resources. Research aimed at understanding the origins of negative cancer beliefs, and identifying ways to improve smokers' engagement with cancer control and health services more widely, may provide the foundations from which to increase the reach of supported smoking cessation programmes.

With regard to early detection, the finding that smokers were as likely to believe that prompt presentation increases the chances of survival (97%), despite their otherwise pessimistic outlook, is interesting. Perhaps although they are aware of the benefits of early detection in principle, this stands against a high perceived likelihood of a bad outcome, and therefore it is not enough to motivate action. In support, Sach and Whynes (2009) found that at the same time as feeling at greater risk of cancer, smokers were less enthusiastic about the offer of colorectal cancer screening. Their reluctance to face up to the imminent risk may undermine motivation towards early diagnosis.

Our analyses benefit from a large, non-clinical sample of older adults, for whom early-detection messages are especially important because both age and smoking duration increase cancer risk. The proportion of current-smokers in our sample (15%) is as expected for this age group (ONS, 2011). However, we cannot assume younger smokers would hold the same beliefs, and studies of younger adults may help to understand smokers' lower participation in cervical screening. Although it was our intention to describe smokers' beliefs about cancer outcomes generally, it is possible that they thought predominantly about lung cancer in their responses due to their greater risk of this disease. Future studies asking questions across a range of different cancer types might help to answer this question. We also cannot presume that these beliefs are markers of stable attitudes because this was a cross-sectional study. To reduce burden we used single-item measures, and the results need to be replicated using more complex measures. Furthermore, although we speculate that pessimistic attitudes could help to explain smokers' lower participation in early detection, studies with behavioural outcomes are needed.

We provide preliminary evidence that smokers hold more negative and avoidant perceptions of cancer outcomes and symptomatic help-seeking, which could underlie their poorer participation in early detection despite their increased risk. Further work is needed to understand smokers' perceptions of different cancer types and how these might relate to engagement with cancer screening. An understanding of the origins of these beliefs is also needed, with potential avenues of study including the effects of tobacco dependence, the extent to which smokers' negativity is socially constructed or consistent across different cultures, and the influence of cognitive dissonance processes. Longitudinal designs that observe the transition in beliefs during changes in smoking status may provide useful insights, as might qualitative study designs that explore smokers' perceptions in depth. This research agenda could help to inform the design of interventions to reduce fatalism and avoidance, and ultimately increase opportunities for earlier diagnosis as well as smoking cessation advice.

## Figures and Tables

**Table 1:** Demographic and health characteristics by smoking status % (*n*)

	**Never-smokers (** ***n*** **=3179)**	**Current-smokers (** ***n*** **=1047)**	**Former-smokers (** ***n*** **=2736)**
**Gender**			
Male	30.4 (968)^a^	39.5 (414)^a^	45.7 (1251)^a^
Female	69.6 (2211)	60.5 (633)	54.3 (1485)
**Age**			
50–59	36.5 (1159)^a^	42.0 (440)^a^	27.3 (746)^a^
60–69	33.8 (1073)	38.5 (403)	38.7 (1058)
70+	29.4 (936)	19.5 (204)	33.9 (927)
Missing	0.3 (11)	0.0 (0)	0.2 (5)
**Marital status**			
Married/cohabiting	56.6 (1799)^a^	45.9 (481)^a^	55.0 (1505)^a^
Single/divorced/separated/widowed	42.9 (1364)	53.5 (561)	44.5 (1218)
Missing	0.5 (16)	0.5 (5)	0.5 (13)
**Ethnicity**			
White	97.5 (3101)^b^	98.7 (1033)^b^	98.4 (2693)^b^
Not White	2.2 (70)	1.1 (11)	1.3 (36)
Missing	0.3 (8)	0.3 (3)	0.3 (7)
**Education Level**			
Left school age ⩽15	25.6 (814)^a^	39.4 (412)^a^	33.4 (913)^a^
CSEs, O-levels or equivalent	21.8 (694)	18.7 (196)	20.6 (563)
A-levels, further education or equivalent	23.9 (760)	23.0 (241)	23.0 (628)
Degree	26.1 (830)	15.5 (162)	21.1 (577)
Missing^c^	2.5 (81)	3.4 (36)	2.0 (55)
**Health status**			
Good	75.8 (2411)^a^	59.2 (619)^a^	67.1 (1837)^a^
Fair	18.7 (596)	27.5 (288)	23.3 (637)
Poor	5.2 (165)	13.3 (139)	9.1 (250)
Missing	0.2 (7)	0.1 (1)	0.4 (12)
**Region**			
England	32.0 (1016)^a^	30.7 (321)^a^	37.3 (1020)^a^
Wales	31.6 (1004)	37.1 (388)	33.1 (906)
Northern Ireland	36.5 (1159)	32.3 (338)	29.6 (810)
**Cancer experience**			
None	19.3 (614)^b^	21.8 (228)^b^	19.3 (527)^b^
Self	7.1 (225)	4.9 (51)	8.1 (221)
Someone close	68.0 (2161)	67.9 (711)	65.1 (1782)
Both	5.3 (168)	5.0 (52)	7.0 (192)
Yes, but would prefer not to say who	0.2 (7)	0.3 (3)	0.2 (5)
Missing	0.1 (4)	0.2 (2)	0.3 (9)

Note: percent totals may not sum due to rounding.

a *χ*^2^, *P*<0.001.

b *χ*^2^, *P*<0.01.

c Includes ‘other' education responses which were not defined.

**Table 2:** Frequencies and multivariable logistic regression models^a^ predicting agreement (agree or strongly agree) with each cancer belief item

	**Never-smokers (reference)**		**Current-smokers**				**Former-smokers**			
	**Agree % (*****n***)	**OR**	**Agree % (*****n***)	**OR**	**95% CI**	**Sig.**	**Agree % (*****n***)	**OR**	**95% CI**	**Sig.**
Going to the doctor as quickly as possible after noticing a symptom of cancer could increase the chances of surviving	97.9 (3113)	1.00	97.0 (1016)	0.66	0.41–1.06	0.084	98.5 (2695)	1.38	0.91–2.11	0.134
Cancer can often be cured	90.6 (2878)^b^	1.00	86.8 (908)	0.73	0.58–0.92	0.008	89.8 (2454)	0.95	0.79–1.14	0.593
These days, many people with cancer can expect to continue with normal activities and responsibilities	90.4 (2867)^c^	1.00	81.5 (853)	0.54	0.44–0.67	<0.001	88.1 (2406)	0.87	0.73–1.04	0.129
Most cancer treatment is worse than the cancer itself	50.9 (1614)	1.00	53.5 (558)	0.99	0.85–1.15	0.926	49.4 (1348)	0.96	0.86–1.07	0.962
A diagnosis of cancer is a death sentence	23.5 (746)^c^	1.00	33.7 (352)	1.55	1.32–1.82	<0.001	23.9 (651)	1.03	0.91–1.17	0.660
I would not want to know if I have cancer	11.3 (359)^c^	1.00	18.0 (188)	1.44	1.17–1.78	0.001	11.4 (311)	0.93	0.78–1.11	0.410

Abbreviations: % CI=95% confidence interval; OR=Odds ratio. Note: reference outcome for logistic regression analyses is those answering disagree or don't know; smoking status is included as an independent variable

a Adjusted for gender, age, marital status, ethnicity, region, education, self-rated health, and cancer experience.

b *χ*^2^, *P*<0.01.

c *χ*^2^, *P*<0.001.

**Table 3:** Frequencies and multivariable logistic regression models^a^ predicting answering yes (often or sometimes) with each barrier to help-seeking item

	**Never-smokers (reference)**		**Current-smokers**				**Former-smokers**			
	**Yes % (n)**	**OR**	**Yes % (*****n***)	**OR**	**95% CI**	**Sig**.	**Yes % (*****n***)	**OR**	**95% CI**	**Sig.**
I would be worried about wasting the doctor's time	33.2 (1056)	1.00	34.9 (365)	1.00	0.86–1.17	0.987	33.3 (912)	1.06	0.94–1.19	0.332
I would be worried about what the doctor might find	28.1 (894)^b^	1.00	35.6 (372)	1.25	1.07–1.47	0.005	27.8 (760)	1.01	0.90–1.14	0.833
I am too busy to make time to go to the doctor	23.4 (744)	1.00	21.4 (224)	0.91	0.76–1.10	0.336	20.3 (556)	0.99	0.87–1.13	0.884
I would be too embarrassed	17.8 (567)^b^	1.00	17.1 (179)	0.79	0.65–0.97	0.022	14.1 (387)	0.81	0.69–0.93	0.004

Abbreviations: % CI=95% confidence interval; OR=Odds ratio. Note: reference outcome for logistic regression analyses is those answering no or don't know; smoking status is included as an independent variable.

a Adjusted for gender, age, marital status, ethnicity, region, education, self-rated health, and cancer experience.

b *χ*^2^, *P*<0.001.
